# The Effect of Fermented Porcine Placental Extract on Fatigue-Related Parameters in Healthy Adults: A Double-Blind, Randomized, Placebo-Controlled Trial

**DOI:** 10.3390/nu12103086

**Published:** 2020-10-11

**Authors:** Dong Hyun Yoon, Ga-Young Han, Su Seung Hwang, Dong Won Lee, Jin-Soo Kim, Keunwon Kim, Jongbae Kim, Wook Song

**Affiliations:** 1 Health and Exercise Science Laboratory, Institute of Sports Science, Seoul National University, Seoul 08826, Korea; ycool14@snu.ac.kr (D.H.Y.); 0104day@naver.com (G.-Y.H.); duexerss@gmail.com (S.S.H.); liger1987@naver.com (D.W.L.); 2 Exercise Medicine Research Institute, Edith Cowan University, Joondalup, WA 6027, Australia; jinsoo.kim@ecu.edu.au; 3 LG Household & Healthcare Research Park, Seoul 07795, Korea; kwkim0412@lghnh.com (K.K.); jbkim@lghnh.com (J.K.); 4 Institute on Aging, Seoul National University, Seoul 03087, Korea

**Keywords:** daily fatigue, fermented porcine placenta, physical fatigue, inflammatory cytokine, treadmill test

## Abstract

Background: Fatigue is one of the major health conditions induced by excessive stress or abnormal immune function or defective antioxidant systems. Placental extract has been reported to have various effects such as immune modulation and cellular regeneration. Fermented porcine placenta (FPP) is a safe nontoxic material, which is highly valuable as a functional food. The aim of this study was to investigate the anti-fatigue effects of FPP supplementation compared with a placebo product. Methods: In this double-blind, parallel, randomized, and placebo-controlled trial 84 healthy males and females, aged between 30 and 60 years were randomized to 320 mg of FPP once daily or placebo. The main outcome measures included efficacy of fatigue-inducing treadmill exercise on physical fatigue and fatigue-related parameters based on the questionnaire administered. Results: The IL-1β mRNA expression and fatigue severity scale were changed significantly after 8 weeks of treatment with fermented porcine placenta compared with placebo (*p* < 0.05). Cortisol levels were significantly improved in participants younger than 45 years following treatment with FPP compared with placebo. Furthermore, the lactate and myoglobin levels were improved significantly in participants with BMI ≥ 23 kg/m^2^ (*p* = 0.045 and *p* = 0.011, respectively) following treatment with FPP versus placebo. Conclusions: Our study showed that FPP supplementation significantly ameliorated fatigue-related parameters and subjective symptoms in healthy adults. Therefore, our results indicate that FPP supplementation induced anti-fatigue effect by regulating the inflammatory response.

## 1. Introduction

Fatigue is a subjective individual symptom characterized by an overwhelming and sustained tiredness, which decreases the physical and mental effectiveness that is not resolved by rest [1]. In modern society, it has become an occupational risk factor as well as a personal challenge. Although fatigue is a frequent symptom in patients diagnosed with multiple sclerosis [2], cancer [3], and other diseases [4,5], it is also common in the healthy individuals, including 14.3% of male and 20.4% of female subjects [6]. Moreover, it has negative effects on physical and mental activities of daily life, which is closely related to the quality of life [7]. Numerous factors, including interleukin 1 (IL-1), C-reactive protein, and interferon γ levels are associated with fatigue [8].

Among the many other clinically available dietary supplements for reducing fatigue, such as caffeine [9,10], theobromine [11], flavanols [12,13], and taurine [14], the placenta has been widely used and commonly available in the health food market with its extensive use against immune disorders and for wound healing and cellular regeneration [15]. The placenta is a vital barrier in the fetus during the gestation period, contributing growth and development of the fetus via exchange gases, nutrients, and waste with their mother via blood [15,16]. Furthermore, it is a well-known source of bioactive compounds, including growth factors, functional peptides, and hormones for fetus growth and development [17]. Porcine placental extract (PPE) is one of the clinically available dietary supplements [15,18], in common with other placental extracts, known for its effectiveness in preventing bone low, enhancing liver function, and hydrating skin [15,19,20].

Fermentation is one of the oldest methods of food preservation along with drying and salting, and is associated with decent flavor, aroma and texture in food industry [21,22]. Fermented porcine placenta (FPP) is receiving more attention due to its health benefits such as altered protein content via fermentation [23]. In addition, FPP is a safe nontoxic material and is highly sought as a functional food [15]. In animal models of exercise-induced physical fatigue, the intake of FPP has been found to improve fatigue and reduced biochemical parameters, such as lactate, lactate dehydrogenase (LDH), glucose, creatine kinase (CK), blood urea nitrogen (BUN), cortisol, and inflammatory cytokines after treadmill stress test [23]. Moreover, a study by Nam et al. [24] reported that the major bioactive compounds in FPP, such as Glycyl-L-Leucine (Gly-Leu, GL) and L-Leucylglycine (Leu-Gly, LG) elevated nitric oxide (NO) production and inducible nitric oxide synthase expression and increased the activity of superoxide dismutase (SOD) in RAW264.7 macrophages. In case of mouse model, the dipeptides decreased the serum concentrations of IL-1β, IL-6, and NO and improved the levels of SOD and glycogen, while decreasing the levels of lactate dehydrogenase, aspartate transaminase, and alanine transaminase [24]. Although animal studies have demonstrated the potential of FPP as anti-fatigue supplements, no reports have investigated its anti-fatigue effect on humans after a single bout of exhaustive exercise. Consequently, the objective of this study was to elucidate the effect of FPP and its major dipeptides, GL and LG, on fatigue and determine its safety in healthy adults. We hypothesized that an eight-week supplementation with FPP has a positive anti-fatigue effect.

## 2. Materials and Methods

### 2.1. Study Design

The study was conducted as a randomized, double-blind, placebo-controlled, 8-week clinical trial from December of 2016 to November of 2017. A total of four study observations were made: during the screening, before ingestion (baseline), four weeks after ingestion, and eight weeks after ingestion. The measurement site was the Seoul National University (Seoul, Korea). In this study, ethics approval was obtained from the Institutional Review Board of Seoul National University, and each participant provided signed informed consent during enrollment (SNUIRB No. 1609/002-019) (KCT0005372).

### 2.2. Study Sample

This study is to investigate the anti-fatigue effect of FPP on the population group with high fatigue. It has been shown that Korean population over 30 years old have higher fatigue compared to other population groups [25]. We have recruited healthy males and females aged 30 to 60 years who complained of fatigue for the study (Fatigue Severity Scale (FSS) ≥ 27 points).

Eighty-four out of 110 applicants were screened according to inclusion and exclusion criteria. Exclusion criteria were inability to perform treadmill test; increased cardiovascular endurance (up to grade 1 and 2 to 50% of VO_2_max depending on age); cardiovascular disease, endocrine/metabolic disease, chronic lung disease, acute or chronic kidney disease; surgery within 6 months; treatment with any other medication known to affect fatigue; risk of allergy against the test food; pregnancy and lactation during the study; and contraindications for the study.

**Figure 1:**
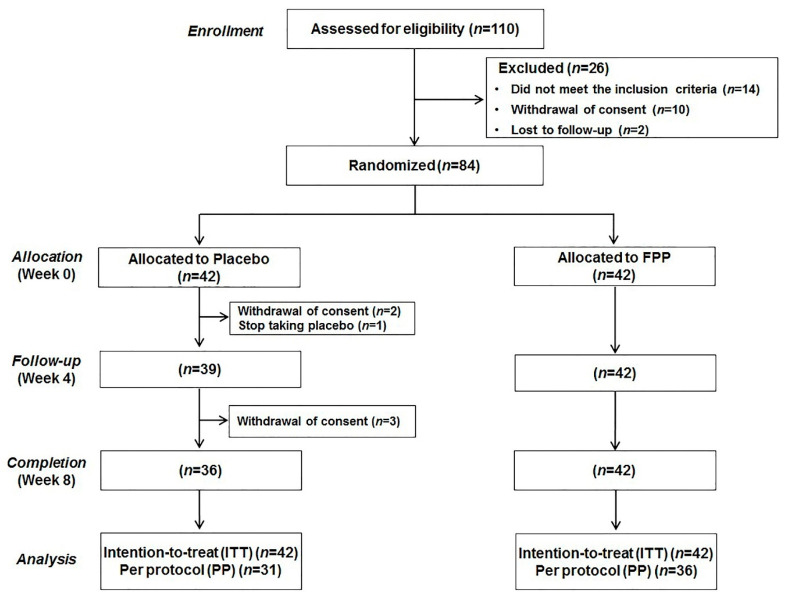
Experimental design.

In this study, subject allocation was made on a 1:1 ratio based on a computer-generated random list (Figure 1). Therefore, subjects were randomly assigned to the placebo and the intervention groups based on gender, age, body mass index (BMI), VO_2_max, and FSS scores. Furthermore, all of these tasks were masked for researchers until all the data were collected and analyzed to minimize bias.

Forty-two subjects were allocated to each group of subjects treated with placebo and a dietary supplement containing FPP. Finally, 67 data with a test compliance greater than 80% and compliant with the guidelines were used in efficacy analysis (Placebo group, 31; FPP group, 36). Table 1 presents the subjects’ baseline characteristics. Subjects consumed four tablets of test food each day with water. During the examination period, meals and exercise, and sleep levels were monitored to ensure appropriate quantity and quality before the start of the study.

### 2.3. Test Product

The test food and placebo were prepared as tablets. Supplements could not be distinguished by appearance, shape or color. Test food tablets contained 80 mg of FPP (Horus Co., Ltd., Tokyo, Japan), while placebo tablets included microcrystalline cellulose instead. The intake of each test sample once daily was 4 tablets; therefore, the intake of FPP was 320 mg (containing 200 μg of dipeptides (Gly-Leu+Leu-Gly)/day). Furthermore, the supplements (placebo or FPP) were distributed to participants after each fatigue-inducing protocol (week 0, week 4), and the compliance was checked at week 4 and the end of experiment.

### 2.4. Measurements

#### 2.4.1. Body Composition

Bioelectrical impendence in individuals was measured using an InBody 720 apparatus analyzer (Biospace Co. Ltd., Seoul, Korea). The participants were asked to fast overnight and engage in normal physical activity. All anthropometric measurements were evaluated by the same person throughout the study to minimize interpersonal variations. The participant’s height was determined using an extensometer, and age, gender, and height were entered into the machine. Once impedance was measured, the results of bodyweight, (BMI), fat mass (FM), fat-free mass (FFM) and percent body fat (% BF) at five different body locations including each arm, each leg, and the trunk as well as a general overall set were acquired [26].

#### 2.4.2. Pre-Testing Protocol

Participant’s maximal exercise capacity was determined by measuring maximal oxygen consumption (VO_2_max) via exercise on a treadmill using T 150 Cosmed and Quark series Breath Pulmonary Gas Exchange for functional diagnostics, until maximal exertion (voluntary cessation). The modified Bruce protocol was used on a treadmill as described in a previous study [25]. To ensure that each subject achieved maximal exertion, at least three of the following four criteria were met by each subject: (1) a plateau in VO_2_ with increasing exercise intensity (<100 mL), (2) a respiratory exchange ratio of at least 1.15, (3) a maximal respiratory rate of at least 35 breaths/min, and (4) a rating of perceived exertion of at least 12 units on the Borg (6–20) scale [25]. 

#### 2.4.3. Fatigue-Inducing Protocol

Fourteen days after their pre-testing, participants returned between 7:00 am and 10:00 am to exercise on the treadmill for 30 min at 70% of their VO_2_max, normally rated as “somewhat hard (12–14 on the scale)” on 6–20 scaled Borg’s perceived exertion scale [27]. Physical fatigue tests were conducted in 0 and 8 weeks.

#### 2.4.4. Primary and Secondary Outcomes

Blood samples were taken at rest, and immediately following exercise cessation, and after 15 and 30 min of recovery to assess physical fatigue. We investigated the serum biochemical profiles, including lactate, (LDH), (CK), myoglobin, TNF-α, SOD, glucose, cortisol, and the serum levels of IL-1β mRNA expression of all the subjects. Participants refrained from consuming nicotine, caffeine, or alcohol, and from vigorous exercise 24 h prior to the exercise test.

Subjective fatigue was evaluated using FSS and Checklist Individual Strength (CIS) based on a total of four measurements: screening, before ingestion (baseline), four weeks after ingestion and eight weeks after ingestion. A recent bibliographic study of fatigue measurement scales suggests that FSS is the most commonly used fatigue specific questionnaire [28]. The FSS is a self-administered questionnaire comprising 9 items (questions) investigating the severity of fatigue in different situations during the past week. Grading of each item ranges from 1 to 7, where 1 indicates strong disagreement and 7 suggesting strong agreement, with the final score representing the mean value of the 9 items. The CIS was developed as a self-reported multidimensional instrument to assess four qualitatively different and relevant aspects of fatigue: fatigue severity (subjective experience of fatigue), concentration problems, reduced motivation, and reduced activity level [29]. It consists of 20 items (questions), each graded 1 to 7, where 1 indicates strong disagreement and 7 strong agreement. The items were designed to measure these dimensions of fatigue.

General biochemical examination of blood and hematologic tests were performed for safety evaluation. Fasting (>12 h) blood and urine samples were collected at baseline and after 8 weeks of intervention. Biochemical parameters were the following: white blood cell count (WBC), red blood cell count (RBC), hemoglobin amount (Hb), hematocrit (Ht), mean red blood cell volume (MCV), mean red blood cell hemoglobin (MCH), mean red blood cell hemoglobin concentration (MCHC), platelet count (PLT), aspartate aminotransferase (AST), alanine aminotransferase (ALT), glucose, creatinine, (BUN), sodium (Na), potassium (K), protein, glucose, ketone, bilirubin, urobilinogen, nitrite, leukocyte esterase, blood color, and pH. The safety of the supplement and the assessment of adverse symptoms were examined via blood and urine analyses, the responsible physician’s inquiries, and subjects’ daily diaries.

The subject’s physical activity levels and diet were measured as possible covariate. Physical activity level was assessed in week 4 and week 8 using International Physical Activity Questionnaire (IPAQ) [30]. Furthermore, dietary intake was assessed for 3 days before testing using a 24-h recall method, and protein intake (g), carbohydrate intake (g), and total calories (kcal) were assessed.

### 2.5. Statistical Analyses

To calculate the sample size, we have used the data from two previous studies, one investigating the effect of the experimental supplement on serum lactate level after treadmill exercise [31], two investigating FSS score after ingesting experimental supplement and placebo [32]. In Leelarungrayub et al. study [31], the alteration of lactate in the experimental group and placebo group was −1.11 mmol/L and 0.12 mmol/L, respectively. We anticipated −1.23 ± 1.72 mmol/L change based on this data after 8 weeks of intervention. To achieve a 90% power and an alpha level of 0.05 (two-tailed), we calculated that 31 subjects are required for each group. Furthermore, based on Zifko et al. study [32], we anticipated −4.30 ± 5.85 changes after 8 weeks of intervention, we calculated that 30 subjects are required for each group. As a result, considering 25% drop-out rate, a total of 84 subjects were recruited and a total of 67 subjects were analyzed (FPP group = 36, Placebo group = 31).

Statistical analyses were performed using SAS version 9.4 (SAS Institute, Cary, NC, USA). Basic characteristics of the study sample were stratified according to treatment with FPP supplement and Placebo and compared using the x^2^ test (categorical variables) and Student’s t test (continuous variables). A linear mixed-effects model including all time points (rest to 30 min; group and time as fixed factors and subject as a random factor) was used to determine the treatment effects. The efficacy assessment was performed via a per-protocol set (PPS) analysis. Exclusion criteria for the PPS analysis were unexpected events or actions affecting fatigue measurement or failure to participate on the designated date and time due to personal circumstances, or test compliance below 80%. Safety assessments were conducted via intent-to-treat (ITT) analysis. Data are shown as the least squares means (LSmean) ± standard error (SE). Threshold for statistical significance was considered at a *p*-value < 0.05.

## 3. Results

Table 2 presents the results of blood lactate levels before and 30 min after recovery following treadmill exercise. The overall lactate level of area under the curve (AUC), the lactate level of the AUC during the fatigue load due to treadmill exercise (pre-peak AUC), and the lactate level of the AUC post-exercise until the recovery phase (post-peak AUC) were analyzed. FPP supplementation demonstrated a decreased tendency in pre-peak AUC (*p* = 0.074).

The IL-1β mRNA expression showed significant group × time interaction after 8 weeks of treatment (*p* = 0.005). However, no significant changes (time × group interaction; *p* > 0.05) in IL-1β mRNA expression were observed between the FPP group and placebo groups at week zero (Table 3).

Table 4 shows the LSmean ± SE values representing changes in FSS pre- and post-supplementation. The internal consistency for FSS and CIS questionaries was tested, and Cronbach’s Alpha was 0.916 and 0.891, respectively. There was no difference in the total FFS score between the groups in baseline (43.5 ± 1.5 vs. 40.3 ± 1.5, *p* = 0.134). However, after the 8 weeks of supplement intakes, significant differences were found between the FPP and placebo groups in changes of total FSS scores (*p* = 0.046), score for the question “my motivation is lower when I am fatigued” (*p* = 0.023), and “fatigue interferes with my physical functioning” (*p* = 0.03). Furthermore, FPP ingestion reduced total FFS score by 9.15 ± 1.27 at 4th week and 9.79 ± 1.28 at the 8th week compared to baseline, whereas only 4.34 ± 1.38 at 4th week and 5.20 ± 1.38 at 8th week were reduced in placebo group. Similarly, the score for the question “my motivation is lower when I am fatigued” showed larger changes overtime point in the FPP group (baseline vs. 4th week, −0.98 ± 0.16; baseline vs. 8th week, −1.30 ± 0.20) compared to the placebo group (baseline vs. 4th week, −0.19 ± 0.18; baseline vs. 8th week, −0.43 ± 0.21). In addition, the score for the question “fatigue interferes with my physical functioning” also showed larger changes overtime points in the FPP group (baseline vs. 4th week, −1.11 ± 0.16; baseline vs. 8th week, −1.33 ± 0.20) compared to the placebo group (baseline vs. 4th week, −0.52 ± 0.18; baseline vs. 8th week, −0.79 ± 0.22) (Table 4). Lastly, the CIS subscale used to measure the subjective experience of fatigue showed a tendency to decrease after eight weeks of ingestion in the FPP intervention group (*p* = 0.073). A significant improvement was observed in items including “physically, I feel exhausted” (*p* = 0.021), and “I feel weak” (*p* = 0.019) (Table A1).

To identify the subjects who showed amelioration of fatigue following FPP supplementation, we conducted subgroup analyses according to subject age (30–44 years vs. 45–60 years) and BMI (<23 kg/m^2^ vs. ≥23 kg/m^2^) (Table 5 and Table 6). Cortisol post-AUC area was improved significantly in subjects younger than 45 years (*p* = 0.017) following supplementation with FPP compared with placebo (Table 5). Furthermore, the lactate and myoglobin levels were improved significantly in subjects whose BMI was ≥ 23 kg/m^2^ (*p* = 0.045 and *p* = 0.011, respectively) upon treatment with FPP versus placebo. No statistically significant differences were detected in the MDA AUC between the FPP and placebo groups based on subgroup analysis according to BMI (Table 6).

Over the course of supplements periods, there were 25 supplement-unrelated minor health-related events (11 in the FPP group, and 14 in the placebo group) confirmed by the physician in charge. These minor events disappeared after a short period, and the supplements were continued for the subjects. Furthermore, all the subjects’ safety parameters, such as vitals, hematology, and blood biochemistry, remained in the physiologically normal range (Table A2).

## 4. Discussion

To our knowledge, this is the first study to analyze the effects of FPP supplementation on the time to fatigue recovery in healthy adults after a single session of fatigue-inducing treadmill test. Based on the physiological properties and beneficial anti-fatigue and performance effects of FPP [16,19,33,34,35], we hypothesized that FPP supplementation enhances recovery from exercise-induced fatigue by improving immune modulation, cellular regeneration, anti-inflammatory cytokine levels, and physiological responses. Moreover, FSS is a validated questionnaire used to evaluate subjective fatigue-related symptoms [36,37]. In this study, the changes in FSS total score decreased significantly in the FPP group compared with the placebo group after 8 weeks of ingestion. The study implied that the anti-fatigue effects of FPP were controlled by the levels of pro-inflammatory cytokines, in accordance with previous animal experiments [23]. As hypothesized, we observed that an 8-week FPP supplementation alleviated the fatigue symptoms in the participants without serious supplement-related side effects.

The double-blind, randomized, placebo-controlled study minimized the chance of bias in sample selection or interpretation of results. We matched the FPP and placebo groups for age, sex, body composition, lifestyle, and cardiopulmonary function. Fatigue is a common symptom in various inflammatory disorders including a variety of illnesses, and was correlated with high levels of inflammatory cytokines (TNF-α, IL-1, and IL-6), which generate fatigue or other symptoms [38]. It has been suggested that pro-inflammatory cytokines, especially interleukin-1 alpha (IL-1α) and interleukin-1 beta (IL-1β), play a significant role in fatigue induction. Several studies have been performed to explain the association between inflammation and these central processes. In particular, IL-1 is of great interest because of its important role in congenital immune system and other physiological systems. Studies reported the role of two of the 11 members belonging to the IL-1 family, including IL-1α and IL-1β in fatigue [39,40,41] In this study, we have shown that 8-week FPP supplementation improves the level of IL-1β. Therefore, it is suggested that the anti-fatigue effect of FPP is mediated via regulation of inflammation, and the anti-fatigue effects result via downregulation of metabolite accumulation in exercise-induced fatigue.

A comprehensive subgroup analysis of the participants according to age (30 to 44 years vs. 45 to 60 years) and BMI (<23 kg/m^2^ vs. >23 kg/m^2^) levels was conducted. Current studies report that subjects within the age range of 30 to 40 years generally showed a higher fatigue index than the older age group [42]. Therefore, our subgroup analysis was consistent with the overall result. Serum cortisol levels after 8 weeks of treatment were significantly reduced in subgroups of subjects below age 45 (*p* = 0.017). However, in the opposite subgroup, it did not significantly inhibit fatigue-inducing serum cortisol levels. Cortisol is a known biomarker of stress induced by physical or psychological stimuli, and extensive measurements have been conducted to assess physical stress response to strenuous exercise [43]. In our subjects, serum cortisol levels increased by treadmill exercise were significantly reduced with FPP treatment, similar to a previous study [23]. These findings indicate that suppressing exercise-induced physical stress with supplements might effectively alleviate fatigue. Our results suggest that the anti-fatigue effect of FPP may be mediated via regulation of serum cortisol levels in human blood.

In the present study, we demonstrated that an 8-week supplementation of FPP was effective in reducing lactate and myoglobin levels in subgroups with BMI 23 kg/m^2^ or higher, leading to protective effects against physical fatigue. These results are similar to the NHANES III study, which reported that groups with higher fatigue had higher BMI in a cross-section of 3125 adults aged 20 to 59 years, and in this study, the anti-fatigue effect of FPP supplement occurred in groups of subjects with BMI greater than 23 kg/m^2^ [44]. However, the effects of FPP on humans have yet to be investigated. Our findings are in agreement with a study conducted by Kim et al. [23], which investigated the effects of FPP powder supplementation on fatigue stress in mice exposed to exercise-induced physical stress. The findings showed that FPP prevented physical fatigue stress by decreasing fatigue-related biochemical parameters (lactate and myoglobin) and increasing the expression of anti-fatigue molecules (SOD, CAT, and glycogen). Lactate is synthesized from pyruvate, mostly during glucose catabolism via the glycolytic pathway or the breakdown of certain amino acids. Under normal aerobic conditions, pyruvate is transported into mitochondria, where it is converted to acetyl-CoA by the pyruvate dehydrogenase (PDH) complex. However, a reduction in PDH enzymatic activity may lead to accumulation of pyruvate and thereby result in excessive lactate synthesis, despite adequate oxygen levels [45].

The improved anti-fatigue effects of FPP supplementation may be explained by the levels of bioactive peptides, such as Gly-Leu and Leu-Gly as demonstrated in the present study [24]. We hypothesize that FPP supplementation increased fatigue-related anti-inflammatory activity mediated by IL-1β, lactate and myoglobin activation; however, additional evidence based on clinical trial studies is needed to corroborate this hypothesis. The changes in FSS total score indicated that FPP alleviated fatigue in subjects regardless of age or other characteristics. In addition, according to the subgroup analysis, the group with higher fatigue level tended to be positively affected by the consumption of FPP.

However, the limitation lies with interpreting the FSS and CIS questionnaires in this study. Although FSS and CIS questionnaires are known and used as reliable tools to assess subjective fatigue for individuals [37,38], recent studies have reported that individual’s subjective feeling of fatigue may be influenced by multiple factors. For instance, a study by Loy et al. [46] and Boolani et al. [47] reported that feeling of fatigue is associated with psychological status, sleep quality as well as a level of mental working [47,48]. However, the current study was conducted without considering those factors (e.g., psychological status, sleep quality, and level of mental working). Only limited interpretation of the FSS and CIS questionnaire results in this study could be made regarding the effect of FPP on fatigue. Although this study assessed the biomarkers for fatigue in serum (e.g., IL-1β mRNA and cortisol) to provide the objective measures for fatigue, further researches considering psychological factor are necessary to understand the effect of FPP on fatigue fully. Furthermore, because CIS also measures participants’ subjective feelings in strength, muscle function test (e.g., 1RM strength test, physical function test) may be necessary to provide further in-depth insight into FPP’s role in muscle function or strength.

## 5. Conclusions

This study is the first clinical trial demonstrating the role of FPP as an anti-fatigue supplement and demonstrated that ingestion of 320 mg/day FPP (containing 200 μg of Gly-Leu and Leu-Gly) significantly ameliorated fatigue-related biochemical parameters and improved fatigue measurement scales in healthy adults. Our results indicate that FPP supplementation induced the anti-fatigue effect via the regulation of inflammatory responses and showed the potential uses of FPP for improving daily life fatigue. However, this study did not examine the potential additive effect of FPP on regular exercise (resistance exercise or aerobic exercise). Investigating the potential additive effect of FPP to improve exercise performance or recovery may be the future direction in a study for FPP. 

## Figures and Tables

**Table 1:** Baseline characteristics of subjects ^1^.

	FPP *n* = 36 (42) ^3^	Placebo *n* = 31 (42) ^3^	*p*-Value ^2^
Age, mean ± SE	44.4 ± 1.3 (43.5 ± 1.3)	41.3 ± 1.5 (41.7 ± 1.2)	0.113 (0.311)
Female, *n* (%)	32 (88.9%) (35 (83.3%))	26 (83.9%) (35 (83.3%))	0.723 (0.100)
BMI (kg/m^2^)	22.8 ± 0.4 (23.1 ± 0.4)	22.3 ± 0.5 (22.5 ± 0.4)	0.400 (0.225)
Alcohol drinker (Y/N)	16/20 (21/21)	13/18 (23/19)	1.000 (0.662)
Alcohol amount (g/week)	12.8 ± 3.8 (22.6 ± 7.7)	14.3 ± 6.0 (24.2 ± 7.8)	0.955 (0.707)
Recommended Food Score	22.8 ± 1.2 (23.9 ± 1.2)	25.4 ± 1.5 (25.5 ± 1.1)	0.141 (0.309)
Fatigue Severity Scale	43.5 ± 1.5 (42.8 ± 1.5)	40.3 ± 1.5 (42.5 ± 1.3)	0.134 (0.877)
VO_2_max (mL/kg/min)	31.6 ± 0.8 (32.2 ± 0.9)	33.3 ± 0.9 (33.5 ± 0.8)	0.165 (0.213)

^1^ Mean ± SE (all such values). FPP, fermented porcine placenta; BMI, body mass index. ^2^ Student’s t-test for continuous variables and Chi-square or Fisher’s exact test for categorical variables were used to compare the difference between the groups. ^3^ The numbers in parentheses indicate the mean and standard error of each group after random allocation.

**Table 2:** Fermented porcine placenta effects for the lactate between groups ^1^.

Variables	FPP Mean ± SE			Placebo Mean ± SE			*p*-Value ^2^		
							Group	Time	Group × Time
AUC (mg/dL × min)									
Week 0	1103.1 ± 79.9			1105.6 ± 85.0					
Week 8	1064.6 ± 79.2			1177.8 ± 85.4			0.573	0.779	0.322
Pre-peak AUC (mg/dL × min)									
Week 0	473.0 ± 64.7			379.6 ± 68.7					
Week 8	370.0 ± 64.1			462.9 ± 69.0			0.998	0.857	0.074
Post-peak AUC (mg/dL × min)									
Week 0	639.3 ± 51.1			726.3 ± 54.2					
Week 8	691.1 ± 50.6			707.8 ± 54.5			0.367	0.739	0.462

^1^ LSmean ± SE (all such values). FPP, fermented porcine placenta; AUC, area under the curve; pre-peak AUC, AUC before peak concentration; post-peak AUC, AUC after peak concentration. ^2^ Linear mixed-effect model adjusted for dietary intake and physical activity (MET) was used to analyze group, time and group × time for 8 weeks.

**Table 3:** Fermented porcine placenta effects for the IL-1β mRNA expression between groups ^1^.

	FPP Mean ± SE	Placebo Mean ± SE	*p*-Value ^2^		
			Group	Time	Group × Time
Week 0					
Resting state	1.60 ± 0.11	1.74 ± 0.12			
End of exercise	1.62 ± 0.11	1.80 ± 0.12			
Recovery 30 min	1.74 ± 0.11	1.95 ± 0.12	0.256	0.001	0.755
Week 8					
Resting state	1.77 ± 0.10	1.62 ± 0.11			
End of exercise	1.72 ± 0.10	1.64 ± 0.11			
Recovery 30 min	1.79 ± 0.10	1.90 ± 0.11	0.780	0.004	0.005

^1^ LSmean ± SE (all such values). FPP, fermented porcine placenta; IL-1β, interleukin-1β. Relative value of IL-1β mRNA expression compared to β-actin (×10^3^). ^2^ Linear mixed-effect model adjusted for dietary intake and physical activity (MET) was used to analyze group, time and group × time at week 0 and week 8.

**Table 4:** Changes of Fatigue Severity Scale between groups ^1^.

Variables	FPP Mean ± SE	Placebo Mean ± SE	*p*-Value ^2^		
			Group	Time	Group × Time
Total					
Week 4	−9.15 ± 1.27	−4.34 ± 1.38			
Week 8	−9.79 ± 1.28	−5.20 ± 1.38	0.026	<0.001	0.046
1. My motivation is lower when I am fatigued.					
Week 4	−0.98 ± 0.16	−0.19 ± 0.18			
Week 8	−1.11 ± 0.16	−0.52 ± 0.18	0.008	<0.001	0.023
2. Exercise brings on my fatigue.					
Week 4	−0.72 ± 0.18	−0.23 ± 0.20			
Week 8	−0.59 ± 0.19	−0.09 ± 0.20	0.082	0.028	0.241
3. I am easily fatigued.					
Week 4	−1.04 ± 0.20	−0.52 ± 0.21			
Week 8	−0.94 ± 0.20	−0.67 ± 0.22	0.195	<0.001	0.361
4. Fatigue interferes with my physical functioning.					
Week 4	−1.30 ± 0.20	−0.43 ± 0.21			
Week 8	−1.33 ± 0.20	−0.79 ± 0.22	0.038	<0.001	0.030
5. Fatigue causes frequent problems for me.					
Week 4	−1.04 ± 0.19	−0.86 ± 0.21			
Week 8	−1.31 ± 0.19	−0.89 ± 0.22	0.355	<0.001	0.419
6. My fatigue prevents sustained physical functioning.					
Week 4	−1.12 ± 0.22	−0.44 ± 0.23			
Week 8	−1.10 ± 0.22	−0.43 ± 0.23	0.064	<0.001	0.097
7. Fatigue interferes with carrying out certain duties and responsibilities.					
Week 4	−0.97 ± 0.18	−0.49 ± 0.20			
Week 8	−1.11 ± 0.18	−0.44 ± 0.20	0.071	<0.001	0.058
8. Fatigue is among my three most disabling symptoms.					
Week 4	−0.93 ± 0.21	−0.55 ± 0.22			
Week 8	−1.13 ± 0.21	−0.70 ± 0.22	0.263	<0.001	0.333
9. Fatigue interferes with my work, family, or social life.					
Week 4	−1.04 ± 0.20	−0.64 ± 0.22			
Week 8	−1.18 ± 0.20	−0.66 ± 0.22	0.178	<0.001	0.274

^1^ LSmean ± SE (all such values). FPP, fermented porcine placenta. ^2^ Linear mixed-effect model adjusted for dietary intake and physical activity (MET) was used to analyze group, time and group × time for 8 weeks.

**Table 5:** Effect of fermented porcine placenta supplementation on cortisol according to subgroup ^1^.

	Age 30–44 Years (*n* = 39)					Age 45–60 Years (*n* = 28)				
	FPP *n* = 19	Placebo *n* = 20	*p*-Value ^2^			FPP *n* = 17	Placebo *n* = 11	*p*-Value ^2^		
			Group	Time	Group × Time			Group	Time	Group × Time
Cortisol										
AUC (ng/dL × min)										
Week 0	26.3 ± 1.5	27.0 ± 1.4				24.1 ± 0.8	23.2 ± 0.9			
Week 8	26.0 ± 1.4	28.0 ± 1.4	0.439	0.723	0.457	23.3 ± 0.7	22.8 ± 0.9	0.464	0.473	0.811
Pre-AUC (ng/dL × min)										
Week 0	9.4 ± 3.1	10.9 ± 2.9				8.6 ± 2.1	4.4 ± 2.4			
Week 8	10.3 ± 2.9	6.4 ± 3.0	0.759	0.317	0.070	1.6 ± 2.0	4.6 ± 2.5	0.796	0.167	0.126
Post-AUC (ng/dL × min)										
Week 0	22.3 ± 2.5	18.3 ± 2.4				18.3 ± 1.7	21.1 ± 2.0			
Week 8	18.2 ± 2.4	22.8 ± 2.5	0.913	0.899	0.017	22.8 ± 1.6	20.7 ± 2.0	0.863	0.280	0.193

^1^ LSmean ± SE (all such values). FPP, fermented porcine placenta; AUC, area under the curve; FSS, Fatigue severity scale. ^2^ Linear mixed-effect model adjusted for dietary intake and physical activity (MET) was used to analyze group, time and group × time for 8 weeks.

**Table 6:** Effect of fermented porcine placenta supplementation on FSS and biomarker according to subgroup ^1^.

	BMI < 23kg/m^2^ (*n* = 52)					BMI ≥ 23kg/m^2^ (*n* = 32)				
	FPP *n* = 26	Placebo *n* = 26	*p*-Value ^2^			FPP *n* = 16	Placebo *n* = 16	*p*-Value ^2^		
			Group	Time	Group × Time			Group	Time	Group × Time
Lactate										
AUC (mg/dL × min)										
Week 0	1015.7 ± 98.5	1110.8 ± 84.0				1297.5 ± 136.7	1309.4 ± 182.0			
Week 8	1211.9 ± 97.9	1141.5 ± 85.6	0.917	0.071	0.140	1028.7 ± 133.5	1472.6 ±192.0	0.285	0.604	0.045
Pre-AUC (mg/dL × min)										
Week 0	375.7 ± 84.2	365.4 ± 71.7				647.2 ± 98.1	567.2 ± 129.6			
Week 8	477.3 ± 83.4	475.5 ± 73.2	0.951	0.079	0.937	341.8 ± 95.3	588.8 ± 139.1	0.562	0.114	0.074
Post-AUC (mg/dL × min)										
Week 0	659.9 ± 63.9	727.3 ± 54.2				662.7 ± 79.9	739.8 ± 105.4			
Week 8	743.9 ± 62.9	664.1 ± 55.5	0.923	0.858	0.185	677.0 ± 77.5	884.2 ± 113.4	0.228	0.278	0.337
Myoglobin AUC (ng/mL × min)										
Week 0	2410.7 ± 178.4	2480.1 ± 145.1				2653.9 ± 215.2	2639.9 ± 288.6			
Week 8	2796.1 ± 175.2	2380.4 ± 154.9	0.342	0.363	0.108	2281.5 ± 211.1	3018.7 ± 300.5	0.297	0.981	0.011
MDA AUC (pmol/mL × min)										
Week 0	12,566.5 ± 1697.1	9184.7 ± 1379.7				10346.4 ± 1144.5	9894.4 ± 1504.8			
Week 8	8296.8 ± 1665.1	7282.1 ± 1472.9	0.198	0.048	0.417	8734.7 ± 1107.9	11,354.1 ± 1638.0	0.493	0.948	0.204

^1^ LSmean ± SE (all such values). FPP, fermented porcine placenta; AUC, area under the curve; FSS, Fatigue severity scale; MDA, malondialdehyde. ^2^ Linear mixed-effect model adjusted for dietary intake and physical activity (MET) was used to analyze group, time and group × time for 8 weeks.

## Appendix A

**Table A1:** Checklist Individual Strength between groups ^1^.

Variables	Placebo	FPP	*p*-Value ^2^					
			Group		Time		Group × Time	
Total								
Week 0	76.63 ± 2.78	79.59 ± 2.64						
Week 4	70.19 ± 2.79	66.85 ± 2.59						
Week 8	68.67 ± 2.81	65.69 ± 2.60	0.725		<0.001		0.161	
Fatigue severity items								
Week 0	32.91 ± 1.45	35.28 ± 1.37						
Week 4	29.38 ± 1.45	27.71 ± 1.35						
Week 8	29.50 ± 1.46	28.44 ± 1.35	0.942		<0.001		0.073	
Concentration items								
Week 0	18.75 ± 0.83	18.79 ± 0.78						
Week 4	17.47 ± 0.83	16.17 ± 0.77						
Week 8	16.57 ± 0.84	15.40 ± 0.77	0.383		<0.001		0.434	
Motivation items								
Week 0	13.97 ± 0.68	13.70 ± 0.64						
Week 4	12.99 ± 0.68	12.26 ± 0.63						
Week 8	13.01 ± 0.68	11.93 ± 0.63	0.368		0.008		0.664	
Activity items								
Week 0	11.01 ± 0.56	11.82 ± 0.53						
Week 4	10.34 ± 0.56	10.71 ± 0.52						
Week 8	9.60 ± 0.57	9.92 ± 0.53	0.426		<0.001		0.775	
1. I feel tired								
Week 0	4.58 ± 0.25	4.94 ± 0.24						
Week 4	3.95 ± 0.25	3.69 ± 0.24						
Week 8	3.86 ± 0.26	4.03 ± 0.24	0.749		<0.001		0.206	
2. I feel very active								
Week 0	3.64 ± 0.27	3.57 ± 0.26						
Week 4	3.49 ± 0.27	3.55 ± 0.25						
Week 8	3.60 ± 0.27	3.49 ± 0.25	0.901		0.917		0.910	
3. Thinking requires effort								
Week 0	3.68 ± 0.23	3.51 ± 0.22						
Week 4	3.30 ± 0.24	2.69 ± 0.22						
Week 8	3.10 ± 0.24	2.62 ± 0.22	0.112		<0.001		0.390	
4. Physically, I feel exhausted								
Week 0	3.79 ± 0.27	4.39 ± 0.25						
Week 4	3.48 ± 0.27	2.89 ± 0.25						
Week 8	3.30 ± 0.27	3.06 ± 0.25	0.762		<0.001		0.021	
5. I feel like doing all kinds of nice things								
Week 0	3.14 ± 0.25	3.29 ± 0.23						
Week 4	2.97 ± 0.25	2.92 ± 0.23						
Week 8	3.06 ± 0.25	2.92 ± 0.23	0.954		0.348		0.757	
6. I feel fit								
Week 0	3.78 ± 0.23	3.67 ± 0.21						
Week 4	3.24 ± 0.23	3.39 ± 0.21						
Week 8	3.66 ± 0.23	3.53 ± 0.21	0.896		0.071		0.662	
7. I think I do a lot in a day								
Week 0	3.69 ± 0.22	3.80 ± 0.21						
Week 4	3.53 ± 0.22	3.57 ± 0.20						
Week 8	3.28 ± 0.22	3.39 ± 0.21	0.708		0.064		0.969	
8. When I am doing something, I can keep my thoughts on it								
Week 0	3.66 ± 0.21	3.60 ± 0.20						
Week 4	3.77 ± 0.21	3.34 ± 0.20						
Week 8	3.44 ± 0.21	3.14 ± 0.20	0.240		0.102		0.488	
9. I feel weak								
Week 0	4.28 ± 0.24	4.59 ± 0.23						
Week 4	3.96 ± 0.24	3.34 ± 0.22						
Week 8	3.59 ± 0.24	3.71 ± 0.22	0.813		<0.001		0.019	
10. I think I do very little in a day								
Week 0	3.58 ± 0.24	4.08 ± 0.23						
Week 4	3.40 ± 0.25	3.70 ± 0.23						
Week 8	3.28 ± 0.25	3.14 ± 0.23	0.399		0.008		0.215	
11. I find it easy to concentrate								
Week 0	3.50 ± 0.21	3.53 ± 0.20						
Week 4	3.48 ± 0.21	3.18 ± 0.20						
Week 8	3.32 ± 0.21	3.26 ± 0.20	0.630		0.361		0.539	
12. I feel rested								
Week 0	4.33 ± 0.23	4.71 ± 0.22						
Week 4	3.55 ± 0.23	3.86 ± 0.22						
Week 8	3.86 ± 0.23	3.62 ± 0.22	0.485		<0.001		0.235	
13. It takes a lot of effort to concentrate on things								
Week 0	3.67 ± 0.23	3.72 ± 0.22						
Week 4	3.10 ± 0.23	3.27 ± 0.21						
Week 8	3.13 ± 0.23	2.96 ± 0.21	0.925		0.002		0.625	
14. Physically I am in bad shape								
Week 0	3.62 ± 0.25	3.90 ± 0.23						
Week 4	3.51 ± 0.25	3.29 ± 0.23						
Week 8	3.60 ± 0.25	3.07 ± 0.23	0.556		0.055		0.074	
15. I have a lot of plans								
Week 0	3.79 ± 0.23	3.32 ± 0.22						
Week 4	3.30 ± 0.23	3.02 ± 0.22						
Week 8	3.42 ± 0.23	2.86 ± 0.22	0.093		0.029		0.689	
16. I tire easily								
Week 0	4.43 ± 0.26	5.03 ± 0.24						
Week 4	3.80 ± 0.26	3.69 ± 0.24						
Week 8	3.62 ± 0.26	3.75 ± 0.24	0.480		<0.001		0.115	
17. I get little done								
Week 0	3.74 ± 0.23	3.96 ± 0.22						
Week 4	3.38 ± 0.23	3.42 ± 0.21						
Week 8	3.07 ± 0.23	3.41 ± 0.21	0.416		0.003		0.686	
18. I don’t feel like doing anything								
Week 0	3.39 ± 0.25	3.52 ± 0.24						
Week 4	3.23 ± 0.25	2.74 ± 0.23						
Week 8	2.96 ± 0.25	2.65 ± 0.23		0.377		0.007		0.286
19. My thoughts easily wander								
Week 0	4.24 ± 0.25	4.44 ± 0.24						
Week 4	3.81 ± 0.25	3.67 ± 0.23						
Week 8	3.61 ± 0.25	3.41 ± 0.23		0.854		<0.001		0.521
20. Physically I feel I am in good shape								
Week 0	4.13 ± 0.24	4.08 ± 0.22						
Week 4	3.81 ± 0.24	3.46 ± 0.22						
Week 8	4.10 ± 0.24	3.71 ± 0.22		0.306		0.028		0.539

^1^ LSmean ± SE (all such values). FPP, fermented porcine placenta. ^2^ Linear mixed-effect model adjusted for dietary intake and physical activity (MET) was used to analyze group, time and group × time for 8 weeks.

## Appendix B

**Table A2:** Hematological test ^1^.

	FPP *n* = 42	Placebo *n* = 42	*p*-Value ^2^
White blood cell (10^3^/μL)			
Week 0	5.4 ± 0.2	5.5 ± 0.2	0.929
Week 8	5.5 ± 0.2	5.4 ± 0.2	0.884
*p*-value ^3^	0.937	0.930	
Red blood cell (10^6^/μL)			
Week 0	4.5 ± 0.1	4.6 ± 0.1	0.547
Week 8	4.5 ± 0.1	4.7 ± 0.1	0.171
*p*-value ^3^	0.289	0.581	
Hemoglobin (g/dL)			
Week 0	13.7 ± 0.2	13.5 ± 0.3	0.714
Week 8	13.5 ± 0.2	13.5 ± 0.3	0.961
*p*-value ^3^	0.180	1.000	
Hematocrit (%)			
Week 0	41.3 ± 0.6	41.1 ± 0.7	0.829
Week 8	41.3 ± 0.7	41.5 ± 0.9	0.863
*p*-value ^3^	0.984	0.312	
Platelet (103/μL)			
Week 0	269.3 ± 10.1	280.9 ± 10.9	0.499
Week 8	272.0 ± 10.7	305.2 ± 11.7	0.033
*p*-value ^3^	0.805	0.015	
MCV (fL)			
Week 0	91.1 ± 0.6	89.5 ± 1.0	0.161
Week 8	91.8 ± 0.7	89.2 ± 1.1	0.056
*p*-value ^3^	0.064	0.463	
MCH (pg)			
Week 0	30.1 ± 0.3	29.4 ± 0.4	0.191
Week 8	30.0 ± 0.3	29.1 ± 0.5	0.111
*p*-value ^3^	0.411	0.138	
MCHC (g/dL)			
Week 0	33.0 ± 0.2	32.8 ± 0.2	0.463
Week 8	32.7 ± 0.2	32.6 ± 0.2	0.587
*p*-value ^3^	0.022	0.132	

^1^ Mean ± SE (all such values). FPP, fermented porcine placenta. MCV, mean corpuscular volume; MCH, mean corpuscular hemoglobin; MCHC, mean corpuscular hemoglobin concentration. ^2^ Student’s t-test was used to compare the difference between the groups. ^3^ Paired t-test was used to compare the difference within each group.
